# Clinical Outcomes of Rural Patients with Diabetes Treated by ECHO-Trained Providers Versus an Academic Medical Center

**DOI:** 10.1007/s11606-024-08925-1

**Published:** 2024-07-09

**Authors:** Matthew F. Bouchonville, Larissa Myaskovsky, Yuridia L. Leyva, Erik B. Erhardt, Mark L. Unruh, Sanjeev Arora

**Affiliations:** 1 Department of Internal Medicine, University of New Mexico Health Sciences Center, Albuquerque, NM USA; 2 Center for Healthcare Equity in Kidney Disease, University of New Mexico Health Sciences Center, Albuquerque, NM USA; 3 Department of Mathematics and Statistics, University of New Mexico, Albuquerque, NM USA

**Keywords:** health disparities, rural medicine, telehealth, diabetes

## Abstract

**Background:**

Despite clinical practice guidelines prioritizing cardiorenal risk reduction, national trends in diabetes outcomes, particularly in rural communities, do not mirror the benefits seen in clinical trials with emerging therapeutics and technologies.

**Objective:**

Project ECHO supports implementation of guidelines in under-resourced areas through virtual communities of practice, sharing of best practices, and case-based learning. We hypothesized that diabetes outcomes of patients treated by ECHO-trained primary care providers (PCPs) would be similar to those of patients treated by specialists at an academic medical center.

**Design:**

Specialists from the University of New Mexico (UNM) launched a weekly diabetes ECHO program to mentor dyads consisting of a PCP and community health worker at ten rural clinics.

**Participants:**

We compared cardiorenal risk factor changes in patients with diabetes treated by ECHO-trained dyads to patients treated by specialists at the UNM Diabetes Comprehensive Care Center (DCCC). Eligible participants included adults with type 1 diabetes, type 2 diabetes on insulin, or diabetes of either type with A1c > 9%.

**Main Measures:**

The primary outcome was change from baseline in A1c in the ECHO and DCCC cohorts. Secondary outcomes included changes in body mass index (BMI), blood pressure, cholesterol, and urine albumin to creatinine ratio (UACR).

**Key Results:**

Compared to the DCCC cohort (*n* = 151), patients in the ECHO cohort (*n* = 856) experienced greater A1c reduction (−1.2% vs −0.6%; *p* = 0.02 for difference in difference). BMI decreased in the Endo ECHO cohort and increased in the DCCC cohort (−0.2 vs. +1.3 kg/m^2^; *p* = 0.003 for difference in difference). Diastolic blood pressure declined in the Endo ECHO cohort only. Improvements of similar magnitude were observed in low-density lipoprotein cholesterol in both groups. UACR remained stable in both groups.

**Conclusions:**

ECHO may be a suitable intervention for improving diabetes outcomes in rural, under-resourced communities with limited access to a specialist.

## Introduction

Diabetes is a major public health problem in the United States, affecting more than 37 million people and carrying substantial personal and economic burdens from medical spending and reduced national productivity.^1,2^ Over the past decade, unprecedented advancements in diabetes therapeutics and technologies have demonstrated efficacy in glycemic management and, importantly, reducing the risk of diabetes-related renal and cardiovascular disease. Unfortunately, the benefits observed in clinical trials have not translated to improved glycemic control nationally, where A1c trends have continued to worsen over this period.^3^ Moreover, significant disparities have emerged, where people with diabetes living in rural communities have not experienced the improvements in blood pressure, cholesterol, or diabetes-related mortality experienced by those living in urban regions.^4,5^

The failure of emerging evidence-based therapies to improve glycemic outcomes nationally is partially attributable to therapeutic inertia and suboptimal uptake of emerging best practices across the diabetes care workforce.^6^ In the US, approximately 90% of those living with diabetes are cared for by the primary care workforce, partly due to a progressive decline in the number of diabetes specialists in the face of rising demand.^7,8^ Although this workforce aligns with the Patient-Centered Medical Home model for optimal chronic disease management, many primary care providers (PCPs) lack confidence in facilitating patient behavior change and implementing increasingly complex clinical practice guidelines around diabetes treatment.^9,10^

Project ECHO is an educational intervention that supports the implementation of best practices for chronic disease management through the development of virtual communities of practice, case-based learning, and peer mentorship. We launched a diabetes-specific program (Endo ECHO) in New Mexico in 2014 to mentor PCPs in rural primary care clinics so that patients could receive higher complexity diabetes care in their communities rather than be referred to a specialist.^11^

We previously reported that a single PCP and community health worker (CHW) diabetes care dyad was recruited at each of 10 primary care clinics in rural New Mexico to participate in weekly virtual Endo ECHO sessions.^11^ Those who participated in the program reported significant improvements in self-efficacy around complex diabetes care and a reduced need for specialist referrals.^12,13^ Patients receiving care from ECHO-trained PCPs reported improvements in access and quality of diabetes care, self-care behaviors, and quality of life.^14,15^ Similarly, patients experienced significant A1c reductions with modest differences compared to community peers with respect to achieving an A1c of <8%.^16^ Current clinical practice guidelines, however, advocate for an intensive multifactorial risk reduction strategy beyond A1c lowering, as this form of intervention has been shown to dramatically improve diabetes outcomes, including mortality.^17,18^ Thus, the current study aimed to assess the impact of the ECHO intervention on several risk factors for cardiovascular and renal disease, specifically exploring whether a model of telementorship could achieve similar quality of care in rural communities as that delivered at a specialist-led referral center. We hypothesized that changes in A1c, blood pressure, low-density lipoprotein (LDL) cholesterol, and urine albumin to creatinine ratio (UACR) in rural patients treated by ECHO-trained PCPs would be no different from those observed in patients treated by diabetes specialists at the academic medical center.

## METHODS

### Study Design

We assembled a multidisciplinary panel of specialists from the academic medical center, including an endocrinologist, behavioral health specialist, nephrologist, pharmacist clinician, diabetes educator, CHW trainer, and social worker. We recruited diabetes care dyads at ten rural New Mexico primary care clinics consisting of a PCP (physician, nurse practitioner, or physician associate) and an individual trained in parallel via ECHO to serve as a CHW. Care dyads attended weekly 2-h Endo ECHO sessions, joining a network of peers via a videoconferencing platform. During these virtual sessions, the dyads collaboratively presented patients with complex diabetes over the network to receive evidence-based guidance from peers and the multidisciplinary specialist panel, fostering diverse perspectives and integrating psychosocial and cultural domains of care. Examples of guidance provided during the sessions included strategies for activating behavioral change, overcoming therapeutic inertia, advancing diabetes pharmacotherapies, and multifactorial risk factor reduction per clinical practice guidelines. In addition to the case-based discussions, a brief clinical didactic was delivered during each session by a member of the multidisciplinary specialist panel or by a guest speaker recognized as a leader in the field. Participating PCPs received free, accredited continuing medical education credit for the weekly sessions. A robust CHW ECHO curriculum and treatment protocol were developed to provide support around self-care behaviors for people with diabetes in collaboration with the PCPs as described previously.^11^

Rural participants (Endo ECHO cohort), who were existing patients of the primary care clinics, were recruited to receive their diabetes care from the ECHO-trained PCP and CHW, at their respective clinics. If needed, participating patients would be discussed in a de-identified manner over the Endo ECHO network per the discretion of the care dyads, but to protect privacy, patients did not participate in the virtual Endo ECHO sessions. To further understand the demographic characteristics of patients with complex diabetes in this study, we collected race and ethnicity data from the electronic medical record (EMR), designated by self-report. Eligible patients were aged 18 years or older, could provide informed consent to participate in the intervention and evaluation, and met at least one of the following criteria for “complex diabetes”: diagnosis of type 1 diabetes, type 2 diabetes on insulin therapy, or diabetes of either type with A1c of >9%. Before participation, the study protocol was approved by the Human Research Protections Office under the Declaration of Helsinki.

A “benchmark” comparison group was retrospectively selected from de-identified data from all patients referred to the University of New Mexico Diabetes Comprehensive Care Center (DCCC), a clinic managed under the Endocrinology Division and led by diabetes specialists. Patients in this clinic were referred to the DCCC by their provider for assistance in complex diabetes management. Of all patients referred to this clinic, those meeting the same eligibility criteria as the Endo ECHO cohort during the same time period were included in the comparison group (DCCC cohort).

### Outcome Measures

Using EMR data from the ten rural primary care clinics and a separate EMR system for the DCCC, we assessed changes from baseline in body mass index (BMI), A1c, blood pressure, LDL cholesterol, and UACR [log_2_(UACR + 1) scale] in consented patients in the Endo ECHO cohort and de-identified patients in the DCCC cohort. To be included in the analysis, baseline and follow-up A1c measurements were required. Relative to the recruitment consent date, for any outcome measures, baseline values were the closest to the consent date within 12 months before to 1 month after, and follow-up values were the latest within 6 to 24 months after the consent date. The primary outcome was a change in A1c for both cohorts from baseline to follow-up.

### Statistical Analysis

We compared baseline characteristics between the Endo ECHO and the DCCC cohort. Categorical variables were summarized as frequencies with percentages and compared using the chi-square test or Fisher’s exact test; continuous variables were summarized as medians with interquartile range (Q1, Q3) or means with standard deviations (SD) and compared using Wilcoxon two-sample test or paired *T*-test. We compared cardiorenal risk factor changes from baseline to follow-up between the Endo ECHO and the DCCC cohort. Multiple regression models for outcome variables were adjusted for age, sex, baseline A1c and BMI, baseline risk factor outcome, and interactions with site (Endo ECHO vs. DCCC), followed by backward selection with Bayesian Information Criterion. We calculated the proportions of patients with controlled diabetes [A1c < 8%] and uncontrolled diabetes [A1c > 9%] to quantify how they changed by cohort (Endo ECHO vs. DCCC). We also employed logistic regression models assessing the difference in difference of proportions to determine whether there was a significant proportion change between Endo ECHO and DCCC cohorts.

## RESULTS

### Baseline Characteristics

In total, 856 patients were recruited from the ten rural clinics in the Endo ECHO cohort, and 151 comparators were retrospectively identified from the academic medical center EMR in the DCCC cohort (Table 1). At baseline, the two groups did not vary in age or sex. The Endo ECHO cohort self-identified predominantly as Hispanic/Latino, 59.3% versus 45.7% in the DCCC cohort (*p* < 0.001). Baseline BMI was significantly greater in the Endo ECHO cohort, 32 versus 30 kg/m^2^ in the DCCC cohort (*p* = 0.002). There were also significant differences at baseline for median A1c, which was 10.3% (IQR 8.9–11.7%) in the Endo ECHO cohort versus 9% (IQR 7.6–11.2%) in the DCCC cohort (*p* < 0.001), diastolic blood pressure, which was 80 mmHg (IQR 71–86 mmHg) in the Endo ECHO cohort versus 71 mmHg (IQR 64–79 mmHg) in the DCCC cohort (*p* < 0.001), and LDL cholesterol, which was 93 mg/dL (IQR 71–120 mg/dL) versus 78 mg/dL (IQR 59–105 mg/dL) in the DCCC cohort (*p* < 0.001). Systolic blood pressure and UACR were similar between groups at baseline.

**Table 1:** Baseline Characteristics of Endo ECHO and DCCC Cohorts

	Endo ECHO (*n* = 856)	DCCC (*n* = 151)	*p*-value^*^
Characteristic	Median [Q1, Q3] or *n* (%)	Median [Q1, Q3] or *n* (%)	
Age	55 [46, 63]	54 [41, 63]	0.11
Sex			0.20
Male	388 (45.3)	77 (51.0)	
Female	468 (54.7)	74 (49.0)	
Ethnicity			**<0.001**
Hispanic/Latino	508 (59.3)	69 (45.7)	
Not Hispanic/Latino	217 (25.4)	80 (53.0)	
Language			**<0.001**
English	732 (85.5)	146 (96.7)	
Spanish	122 (14.3)	4 (2.6)	
HbA1c	10.3 [8.9, 11.7]	9.0 [7.6, 11.2]	**<0.001**
BMI (kg/m^2^)	32 (28, 37)	30 [26, 36]	**0.002**
Blood Pressure			
Systolic (mmHg)	130 [119, 142]	128 [118, 140]	0.23
Diastolic (mmHg)	80 [71, 86]	71 [64, 79]	**<0.001**
Cholesterol			
Total	177 [149, 210]	–	–
LDL	93 [71, 120]	78 [59, 105]	**<0.001**
HDL	44 [36, 50]	–	–
Triglycerides	171 [110, 227]	–	–
Urine albumin to creatinine ratio	27.8 [10.0, 110.8]	20.9 [8.3, 61.2]	0.31

^*^Wilcoxon two-sample test for comparison of medians, or chi-square test for categorical variables. Missing values: ethnicity (133), language ^13^

### Outcome Measures

**Figure 1:**
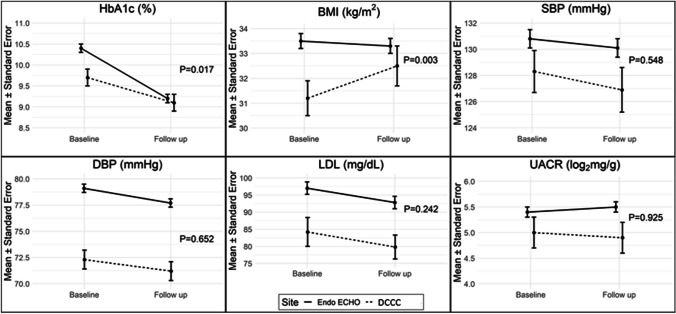
*P*-values represent difference in difference between change in outcomes between Endo ECHO and DCCC cohorts. *P*-values are based on model results, while means and standard errors are based on observed values. BMI, body mass index; SBP, systolic blood pressure; DBP, diastolic blood pressure; LDL, low-density lipoprotein; UACR, urine albumin to creatinine ratio.

The average follow-up duration was 19.5 months in the Endo ECHO cohort and 16.9 months in the DCCC cohort. BMI decreased in the Endo ECHO cohort and increased in the DCCC cohort (−0.2 vs. +1.3 kg/m^2^; *p* = 0.003 for difference in difference) (Table 2 and Fig. 1). The reduction in mean A1c was significantly greater in the Endo ECHO cohort (−1.2 vs. −0.6%; *p* = 0.02 for difference in difference). Though groups were different at baseline, by the end of the study, a similar proportion of patients with diabetes achieved an A1c < 8%: 31.4% of the Endo ECHO cohort and 32.5% of the DCCC cohort (*p* = 0.829). Except for a diastolic pressure reduction in the Endo ECHO cohort (−1.4 mmHg; *p* = 0.010), the trends in blood pressure were non-significant and similar between groups. Compared to baseline, UACR remained stable between groups (*p* = 0.93 for difference in difference). LDL cholesterol declined in the Endo ECHO cohort (−4.2 mg/dL, *p* = 0.046) and in the DCCC cohort (−4.4 mg/dL, *p* = 0.044), with similar changes between groups (*p* = 0.24 for difference in difference). Though comparison data were not available from the DCCC cohort, we did observe a reduction in total cholesterol in the Endo ECHO cohort (−14.6 mg/dL; *p* = 0.036), but no significant changes in HDL cholesterol (+0.9 mg/dL; *p* = 0.938) or triglycerides (−52.8 mg/dL; *p* = 0.080).

**Table 2:** Difference in Difference of Outcomes in Endo ECHO Versus DCCC Cohort from Baseline to Follow-up

Outcome	Endo ECHO (*n* = 856)			DCCC (*n* = 151)			Endo ECHO vs. DCCC (difference in difference)	
	Baseline	Follow-up	Difference	Baseline	Follow-up	Difference		
	Mean (SE)	Mean (SE)	*P*-value^*^	Mean (SE)	Mean (SE)	*P*-value^*^	Effect size^†^	*P*-value^†^
HbA1c (%)	10.4 (0.1)	9.2 (0.1)	<0.001	9.7 (0.2)	9.1 (0.2)	0.042	−0.64 (−1.20, −0.12)	0.02
BMI (kg/m^2^)	33.5 (0.3)	33.3 (0.3)	0.061	31.2 (0.7)	32.5 (0.8)	0.037	−0.94 (−1.60, −0.32)	0.003
SBP (mmHg)	130.8 (0.7)	130.1 (0.7)	0.320	128.3 (1.6)	126.9 (1.7)	0.832	−1.24 (−5.30, 2.80)	0.55
DBP (mmHg)	79.1 (0.4)	77.7 (0.4)	0.010	72.3 (0.9)	71.2 (0.9)	0.769	−0.60 (−3.20, 2.00)	0.65
Total cholesterol (mg/dL)	186.1 (4.9)	171.5 (3.9)	0.036	–	–	–	–	–
LDL (mg/dL)	97.0 (1.8)	92.8 (1.8)	0.046	84.2 (4.2)	79.8 (3.5)	0.044	5.96 (−4.00, 16.0)	0.24
HDL (mg/dL)	44.4 (1.1)	45.3 (1.2)	0.938	–	–	–	–	–
Triglycerides (mg/dL)	232.3 (25.7)	179.5 (9.3)	0.080	–	–	–	–	–
UACR (log_2_mg/g)	5.4 (0.1)	5.5 (0.1)	0.410	5.0 (0.3)	4.9 (0.3)	0.781	0.03 (−0.53, 0.59)	0.93

^*^Paired *T*-test for comparison of means

^†^*P*-values are based on the difference in difference linear regression models adjusted for age, sex, baseline A1c and BMI, baseline risk factor outcome, and interactions with site (Endo ECHO vs DCCC), followed by backward selection with BIC. *UACR*, urine albumin to creatinine ratio; no total cholesterol, HDL, or triglyceride data available in final dataset for cells with “–”; missing values for baseline measures: total cholesterol (878); triglycerides (878); HDL (866); urine albumin to creatinine ratio (555); LDL (457); BMI (261); HbA1c (123); SBP (73); DBP (72). Missing values for follow-up measures: HDL (883); total cholesterol (879); triglycerides (879); LDL (487); BMI (321); HbA1c (244); SBP (164); DBP (163)

We identified significant differences between cohorts in the change in the proportion of patients meeting criteria for “controlled” (A1c < 8%) versus “uncontrolled” diabetes (A1c > 9%), compared to baseline (Table 3). In the Endo ECHO cohort, achievement of an A1c < 8% increased by 20.1% from baseline versus 0.3% in the DCCC cohort (*p* < 0.001 for difference in difference) (Table 3). In contrast, those with an A1c > 9% decreased by 24.8% from baseline in the Endo ECHO cohort and by 4.9% in the DCCC cohort (*p* = 0.002 for difference in difference).

**Table 3:** Changes in the Proportion of Patients Meeting HEDIS Criteria for “Controlled vs Uncontrolled” Diabetes

Proportions of patients with controlled diabetes (A1c < 8%)						Proportions of patients with uncontrolled diabetes (A1c > 9%)				
	Baseline	Follow-up	Difference	Endo ECHO vs. DCCC (difference in difference)		Baseline	Follow-up	Difference	Endo ECHO vs. DCCC (difference in difference)	
				Odds (95% CI)	*p*-value				Odds (95% CI)	*p*-value
Endo ECHO (*n* = 856)	11.3%	31.4%	+20.1%	Endo ECHO, follow-up vs. baseline: 3.60 (2.72, 4.78)	**<0.001**	72.0%	47.2%	−24.8%	Endo ECHO, follow-up vs. baseline: 0.35 (0.28, 0.43)	**<0.001**
DCCC (*n* = 151)	32.2%	32.5%	+0.3%	DCCC, follow-up vs. baseline: 1.01 (0.60, 1.70)	0.97	49.7%	44.8%	−4.9%	DCCC, follow-up vs. baseline: 0.82 (0.50, 1.34)	0.43

*HEDIS* Healthcare Effectiveness Data and Information Set

## DISCUSSION

Mentoring PCPs and CHWs through the ECHO model to build diabetes expertise in rural, medically underserved communities was associated with significant improvements in multiple cardiorenal risk factors, including A1c, blood pressure, and cholesterol among patients with diabetes. Moreover, patients received this care in their communities rather than being referred out to a specialist. Based solely on clinical outcomes in this report, this care was similar to the care provided by diabetes specialists at an academic medical center accepting referrals from the entire state. In an increasingly common landscape where access to a diabetes specialist is scarce and patients face geographic barriers to care, an outreach program such as Project ECHO holds promise for reducing disparities affecting rural patients with diabetes.

The current findings are the first to describe the impact of the ECHO program on cardiorenal risk factor changes in patients with diabetes treated by ECHO-trained providers compared to a benchmark group at an academic medical center. The intervention was piloted in a largely rural and Health Resources and Services Administration–designated medically underserved state with a high prevalence of diabetes and diabetes-related complications. Moreover, robust electronic medical record data permitted an evaluation including hundreds of participants with diabetes in rural communities across New Mexico. The ECHO model of longitudinal telementorship supports implementing best practices through case-based and community-of-practice learning beyond the conventional webinar format of knowledge dissemination.^19^ The multidisciplinary nature of the ECHO sessions fosters a diversity of perspectives and integration of social determinants of health into personalized care plans, which is essential for overcoming clinical inertia. Further, the intervention uses specialist-guided peer mentorship, such that a large workforce of learners can be engaged and upskilled simultaneously. The current findings align with a growing narrative of patient and provider-reported outcomes demonstrating improvements in self-perceived diabetes care through participation in ECHO. Additionally, there are implications around reducing the burden of diabetes-related complications and morbidity when multiple cardiorenal risk factors are improved in a traditionally hard-to-reach population. One recent report of Medicaid claims demonstrated that patients with diabetes treated by ECHO-trained PCPs experienced 44% fewer hospitalizations and 62% lower inpatient spending compared to patients treated by non-participating PCPs.^20^ A growing ecosystem of such programs in the US with a shared mission of reducing diabetes-related health disparities has recently been described and convenes monthly to share best practices and strategize around collaborative initiatives.^21^

There are important limitations to this report, perhaps the most significant of which is the use of a retrospective comparison group consisting of patients that were similar in age and sex but different in terms of ethnicity and several clinical measures at baseline. Additionally, we could not distinguish between type 1 and type 2 diabetes in our dataset. We reached a cohort of predominantly Hispanic rural community members distinct from those typically seen at the academic medical center. Notably, patients referred to the DCCC had lower A1c measures on average at baseline which may have contributed to the more robust A1c lowering in the Endo ECHO cohort. However, despite starting at a higher A1c on average, those in the Endo ECHO cohort achieved similar A1c values to the DCCC cohort by the end of the intervention. Further, those in the Endo ECHO cohort with higher baseline A1c values were more likely to experience improved glycemic control than those with similar baseline values in the DCCC cohort. Because patients were selected for enrollment primarily based on glycemic status, baseline values for blood pressure and cholesterol measures were in a range considered in line with current standards of care, a supported finding for both cohorts. Nonetheless, multifactorial risk reduction was central to the ECHO intervention and small improvements in both measures were observed, which were similar between cohorts. Urine microalbumin values remained stable in both cohorts for the duration of this study. An evaluation designed to evaluate the impact of ECHO on CKD progression would be of great value, particularly as more recent standards of care in diabetes have prioritized early initiation of renoprotective therapies that were less available at the onset of the current study. A further limitation to the study design was that we compared 10 rural primary care clinics to a single specialist-run clinic, resulting in unequal participant group sizes. Although data from both groups were analyzed identically, including only those with post-intervention data may have selected for more compliant experimental groups. Additionally, we do not have data regarding the frequency of visits for rural participants and it is possible that these individuals sought more regular follow-up. Training a diabetes workforce within the communities where patients reside greatly improves access to more regular care versus traveling to a referral center with extensive wait times. Finally, the retrospective nature of the current analysis did not allow for control over follow-up duration, which was slightly longer (2.6 months) in the Endo ECHO cohort than the typical follow-up period in the DCCC. Taken as a whole, we identified no clinical domain signaling inferior outcomes for rural patients who received care by ECHO-trained dyads compared to those referred to the academic medical center.

Despite these limitations, in the current study, we reached a rural population of predominantly non-White individuals with a high prevalence of Medicaid insurance coverage.^22^ Healthcare delivery strategies that build capacity in rural communities may help reduce disparities by optimizing best-practice care in the communities where at-risk individuals live. We used the ECHO model to train a workforce of rural PCPs and CHWs in a state challenged by disproportionate diabetes rates and a shortage of diabetes specialists. We observed that patients treated by ECHO-trained dyads experienced significant improvements in multiple cardiorenal risk factors, which were similar to improvements experienced by patients treated by diabetes specialists at an academic medical center. These findings support the need for prospective, controlled trials evaluating the impact of the ECHO model on clinical outcomes for vulnerable populations with diabetes. Healthcare professionals may consider the ECHO model as a potential solution for overcoming clinical inertia and improving diabetes-related health disparities affecting patients in rural communities with limited access to specialists.

## Data Availability

Some or all datasets generated or analyzed during the current study are not publicly available but are available from the corresponding author upon reasonable request.
